# Supplementary material for: Comparison of Cortisol, Androstenedione and Metanephrines to Assess Selectivity and Lateralization of Adrenal Vein Sampling in Primary Aldosteronism
Source: J Clin Med. 2021 Oct 17;10(20):4755. doi: 10.3390/jcm10204755 (PMC8538328; doi:10.3390/jcm10204755)
Supplement: Supplementary file 1 [file jcm-10-04755-s001.zip › jcm-1399107-supplementary.pdf]

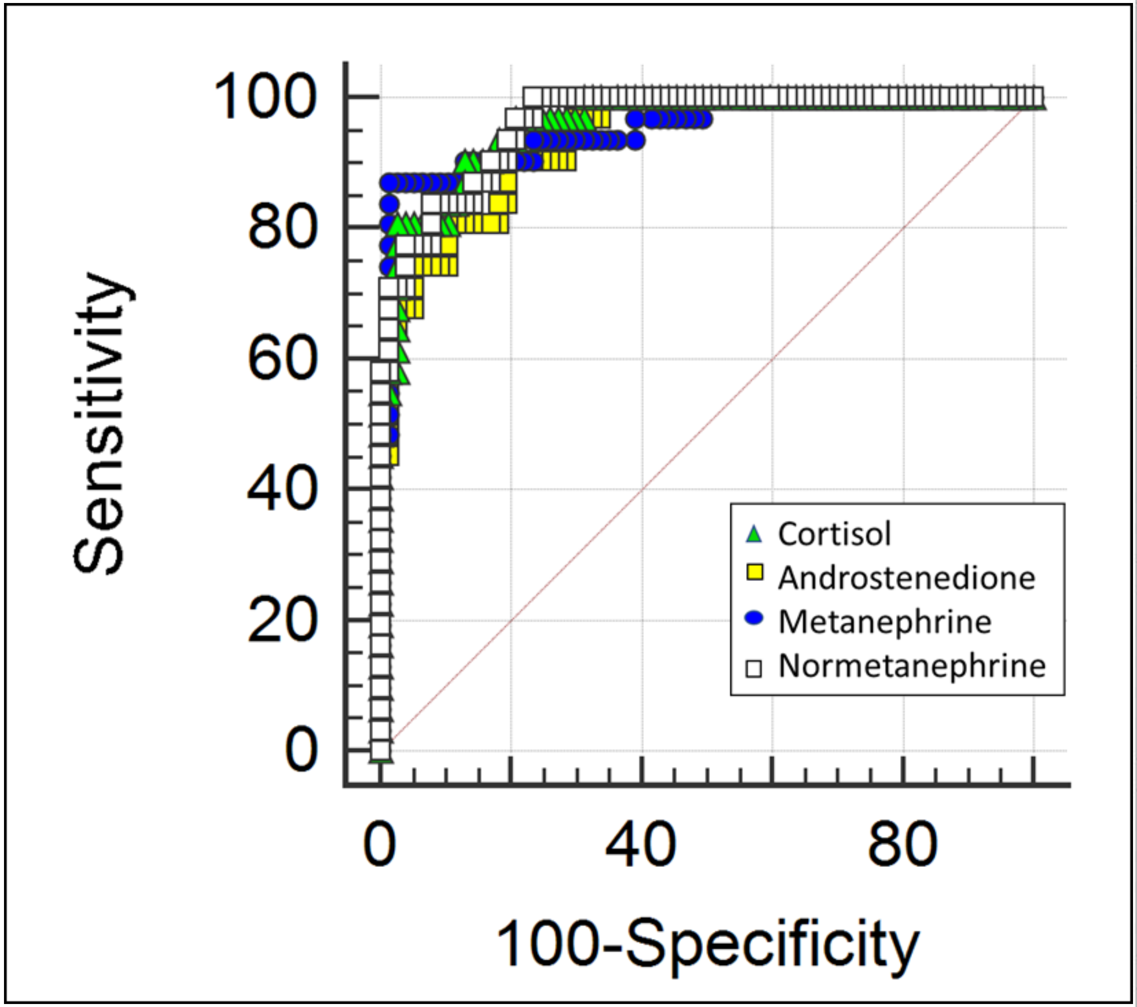

Pairwise comparison of ROC curves

**test_Cort_1 ~ testANDRO_1**	
Difference between areas	0,0222
Standard Error [a]	0,0150
95% Confidence Interval	-0,00727 to 0,0517
z statistic	1,476
Significance level	P = 0,1398
**test_Cort_1 ~ testMETA_1**	
Difference between areas	0,00566
Standard Error [a]	0,0207
95% Confidence Interval	-0,0349 to 0,0462
z statistic	0,273
Significance level	P = 0,7845
**test_Cort_1 ~ testMN_1**	
Difference between areas	0,000419
Standard Error [a]	0,0148
95% Confidence Interval	-0,0285 to 0,0293
z statistic	0,0284
Significance level	P = 0,9773
**testANDRO_1 ~ testMETA_1**	
Difference between areas	0,0165
Standard Error [a]	0,0215
95% Confidence Interval	-0,0256 to 0,0587
z statistic	0,770
Significance level	P = 0,4412
**testANDRO_1 ~ testMN_1**	
Difference between areas	0,0218
Standard Error [a]	0,0178
95% Confidence Interval	-0,0131 to 0,0566
z statistic	1,225
Significance level	P = 0,2207
**testMETA_1 ~ testMN_1**	
Difference between areas	0,00524
Standard Error [a]	0,0167
95% Confidence Interval	-0,0274 to 0,0379
z statistic	0,314
Significance level	P = 0,7534

[a] Hanley & McNeil, 1983

Supplemental Figure S1: Comparison of the Receiver operating characteristic curves (ROC) for the accuracy of lateralization index based on the cortisol, androstenedione, metanephrine and normetanephrine. The area under the curves, an overall measure of accuracy, showed no significant differences across the different curves, thus indicating that use of androstenedione (B), metanephrine (C) and normetanephrine (D) did not worsen the diagnostic accuracy as compared to cortisol.
